# Functional Balancing of the Hypoxia Regulators RAP2.12 and HRA1 Takes Place *in vivo* in *Arabidopsis thaliana* Plants

**DOI:** 10.3389/fpls.2017.00591

**Published:** 2017-04-25

**Authors:** Beatrice Giuntoli, Francesco Licausi, Hans van Veen, Pierdomenico Perata

**Affiliations:** ^1^Plant Lab, Institute of Life Sciences, Scuola Superiore Sant'AnnaPisa, Italy; ^2^Biology Department, University of PisaPisa, Italy

**Keywords:** low oxygen, regulation of anaerobic gene expression, ERF-VII transcription factors, trihelix transcription factor family, transcription factor balancing

## Abstract

Plants are known to respond to variations in cellular oxygen availability and distribution by quickly adapting the transcription rate of a number of genes, generally associated to improved energy usage pathways, oxygen homeostasis and protection from harmful products of anaerobic metabolism. In terrestrial plants, such coordinated gene expression program is promoted by a conserved subfamily of ethylene responsive transcription factors called ERF-VII, which act as master activators of hypoxic gene transcription. Their abundance is directly regulated by oxygen through a mechanism of targeted proteolysis present under aerobic conditions, which is triggered by ERF-VII protein oxidation. Beside this, in *Arabidopsis thaliana*, the activity of the ERF-VII factor RAP2.12 has been shown to be restrained and made transient by the hypoxia-inducible transcription factor HRA1. This feedback mechanism has been proposed to modulate ERF-VII activity in the plant under fluctuating hypoxia, thereby enhancing the flexibility of the response. So far, functional balancing between RAP2.12 and HRA1 has been assessed in isolated leaf protoplasts, resulting in an inverse relationship between HRA1 amount and activation of RAP2.12 target promoters. In the present work, we showed that HRA1 is effective in balancing RAP2.12 activity in whole arabidopsis plants. Examination of a segregating population, generated from *RAP2.12* and *HRA1* over-expressing plants, led to the first quantitative proof that, over a range of either transgene expression levels, HRA1 counteracts the phenotypic and transcriptional effects of RAP2.12. This report supports the occurrence of fine-tuned regulation of the hypoxic response under physiological growth conditions.

## Introduction

Conditions characterized by sub-optimal oxygen levels are considered common in plants (van Dongen and Licausi, 2015). Cellular oxygen concentrations can fall below the threshold set by the mitochondrial complexes for optimal aerobic respiration in several situations, ranging from soil waterlogging, with consequent root asphyxia, and flooding stress (Drew, 1997; Voesenek et al., 2006), to tight packaging of cells inside compact structures like bulky tissues and fruits (Ho et al., 2011; Licausi et al., 2011a), biogenesis of gas-impermeable layers in some seeds (Borisjuk and Rolletschek, 2009), or the existence of underground organs (De Willigen and van Noordwijk, 1989). Terrestrial plants have evolved a wide range of adaptations to either prevent the onset of severe hypoxia in their organs, or improve metabolism during a shortage of oxygen (Bailey-Serres and Voesenek, 2008). On the other hand, in some cases, hypoxia can help cells control the production of dangerous oxidative conditions, so that it becomes even required for the correct progression of specific developmental processes, such as and pollen differentiation inside maize anthers (Kelliher and Walbot, 2012, 2014).

Hypoxic responses have been associated to a wide reconfiguration of plant transcriptomes (Branco-Price et al., 2005; Loreti et al., 2005; Mustroph et al., 2010; Lee et al., 2011). Regulation of transcription upon oxygen deprivation relies on ERF-VII transcription factor family members (Nakano et al., 2006), which redundantly activate the expression of the complete set of hypoxia-responsive genes by direct promoter recognition (Bui et al., 2015; Papdi et al., 2015; Gasch et al., 2016). Over-expression of the RAP2.12 member of the ERF-VII subfamily, for instance, is sufficient to trigger the core transcriptional response to hypoxia in *Arabidopsis thaliana*, as previously defined (Mustroph et al., 2009), even in the absence of the corresponding external stimulus (Licausi et al., 2011b). The activity of RAP2.12 is tightly regulated by oxygen through a targeted proteolytic pathway, whereby the transcription factor is made oxygen-labile, coupled to a subcellular localization mechanism that guarantees the cell the presence of quickly available RAP2.12 as hypoxia arises (Kosmacz et al., 2015). Indeed, RAP2.12 is post-transcriptionally regulated by oxygen through a direct biosensing mechanism that deploys the Cys/Arg branch of the N-end rule pathway for proteasomal degradation (composed, in Arabidopsis, by the arginyl aminotransferase enzymes ATE1 and ATE2 and by the E3 ubiquitin ligase PRT6) and plant cysteine oxidase enzymes (Gibbs et al., 2011; Weits et al., 2014).

Beside the basic working principle of this oxygen biosensor, it has been shown that further mechanisms of regulation exist that empower plant cells to achieve improved control of RAP2.12 activity. In detail, a feedback loop has been described, in which the transcription factor HRA1 can act on RAP2.12 to restrain its transactivation power on target genes (Giuntoli et al., 2014). Intriguingly, the up-regulation of *HRA1* homologs in response to low oxygen in several species allows the attribution of HRA1 to the set of plant core conserved hypoxia-responsive genes (Mustroph et al., 2010). It has been put forward that the induction of HRA1 might be used by plants to produce transient pulses of anaerobic gene expression promoted by RAP2.12, which would enable dynamic and fast regulation in response to conditions of fluctuating hypoxia (Giuntoli et al., 2014). However, an assessment of the influence of this mechanism *in planta* is still needed.

In order to understand whether the interaction of RAP2.12 and HRA1 transcription factors results in a functional balancing in the plant, we decided to study how plant morphology and gene expression are affected when both genes were over-expressed in a constitutive fashion. Our results report that HRA1 had measurable effects on the processes downstream of RAP2.12. Our findings give way to future experiments to gain more in-depth knowledge regarding the range of action of the HRA1 fine-tuning function in the plant.

## Materials and methods

### Generation of Δ13RAP2.12xHRA1 double over-expressors in *Arabidopsis thaliana*

Two stabilized transgenic lines over-expressing the individual genes, isolated in previous works, were crossed (Licausi et al., 2011b; Giuntoli et al., 2014). *35S:HRA1* plants express the coding sequence of *HRA1* fused to a C-terminal FLAG tag sequence, under control of the CaMV 35S promoter. *35S:*Δ*13RAP2.12* plants, instead, encode an N-end rule insensitive version of RAP2.12 lacking the first 13 N-terminal residues. Both lines were generated in the Columbia-0 background. Homozygous parental plants were crossed manually and the hybrid progeny was propagated to the following F_2_ generation.

### Plant growth conditions and sampling

Seeds were sown in a moist mixture of soil perlite 3:1 and stratified at 4°C in the dark for 48 h. Plants were grown at 23°C day/18°C night under a neutral day cycle (12 h light/12 h darkness, ~100 μmol photons m^−2^s^−1^ light intensity). Upon attainment of the developed rosette stage (stage 3.50 Boyes et al., 2001), corresponding to 4–5 weeks of growth in our conditions, plants were evaluated phenotypically and subsequently sampled for gene expression analyses.

### RT-qPCR

Transcript abundance was measured in whole rosettes of stage 3.50 (Boyes et al., 2001) arabidopsis plants, by means of real time quantitative PCR. RNA extraction, removal of genomic DNA, cDNA synthesis and RT–qPCR analyses were performed as described previously (Licausi et al., 2011c). The sequences of the primers used for cDNA amplification are listed in Table 1. Steady-state mRNA levels were normalized using *UBQ10* as the reference gene, and relative expression values were calculated using the comparative Ct method (Schmittgen and Livak, 2008). Total *RAP2.12* and *HRA1* expression was assessed with primers annealing on the respective coding sequences. In non-transgenic plants, total gene expression coincided with the level of the endogenous transcripts encoded by the wild type genome. On the opposite, in transgenic plants, 3′-UTR sequences were exploited in order to discriminate between the expression of transgenes and endogenous genes. Specifically, expression of the *RAP2.12* transgenic sequence (referred to as *transRAP2.12*) was measured directly, through an amplification product spanning over the 3′-UTR region encoded by the transgenic construct. On the other hand, *HRA1* transgene expression (*transHRA1*) was calculated by subtracting the endogenous *HRA1* expression level, measured with specific *HRA1* 3′-UTR genomic primers, from the total amount of *HRA1* transcript, measured with primers annealing on the coding sequence. Transgenic mRNA abundance was subsequently expressed as relative to the level measured in one selected plant from the relative parental line, in which it was set to 100%.

**Table 1:** **Nucleotide sequences of the primers used in the RT-qPCR analyses**.

**Locus name (AGI code)**	**Primer name**	**Primer sequence (5′-3′)**
*ADH1 (At1g77120)*	ADH1_F	tattcgatgcaaagctgctgtg
	ADH1_R	cgaacttcgtgtttctgcggt
*HB1 (At2g16060)*	Hb1_F	tttgaggtggccaagtatgca
	Hb1_R	tgatcataagcctgaccccaa
*HRA1 (At3g10040)*	HRA1_F	tcatgttacggcggagtgaa
	HRA1_R	caacccgtgtacccgaagac
	HRA1_Endo_F	gggaagaagcggcaagtgtagtg
	HRA1_Endo_R	tttactgcctaatgtcactaaaacgtgag
	HRA1_Tot_F	agtcagcagcagaactgttttcacg
	HRA1_Tot_R	tctccactccttcccactcataccc
*HSP18.2 (At5g59720)*	HSP18.2_F	ggcctgaagaaggaagaagtcaagg
	HSP18.2_R	agcacacaagctttttatttgacacacc
*HUP7 (At1g43800)*	HUP7_F	accaatgttggcaacccgcttc
	HUP7_R	tttccctcagctcacgaacctg
*LBD41 (At3g02550)*	LBD41_F	tgaagcgcaagctaacgca
	LBD41_R	atcccaggacgaaggtgattg
*PCO1 (At5g15120)*	PCO1_F	attgggtggttgatgctccaatg
	PCO1_R	atgcatgttcccgccatcttc
*PDC1 (At4g33070)*	PDC1_F	cgattatggcactaaccggatt
	PDC_1R	tgttcaccaccgcctgataac
*RAP2.12 (At1g53910)*	RAP2.12_F	actgaatgggacgcttcactgg
	RAP2.12_R	agggtttgcaccattgtcctgag
	transRAP2.12_F	tgggacgcttcactggatttcc
	transRAP2.12_R	cgcgcccaccctttcagaag
*UBQ10 (At4g05320)*	UBQ10_F	ggccttgtataatccctgatgaataag
	UBQ10_R	aaagagataacaggaacggaaacatagt

### Statistical analysis

In the segregating F_2_ population (*n* = 32), resulting from the cross of *35S:HRA1* and *35S:*Δ*13RAP2.12* parental lines of arabidopsis, the interaction between the two factors under investigation was evaluated upon measurement of the expression level of eight anaerobic marker genes, known from the literature as targets of RAP2.12. Specifically, a linear model was fit to the scatterplot expression of every marker gene, used as output variable, in dependence of two chosen predictors, namely total *RAP2.12* expression values and *transHRA1* presence. The analyses of covariance and linear regressions were performed with the R statistical software (R Development Core Team, 2010).

## Results

### Over-expression of *HRA1* restrains the impact of *Δ13RAP2.12* on plant phenotype

**Figure 1:**
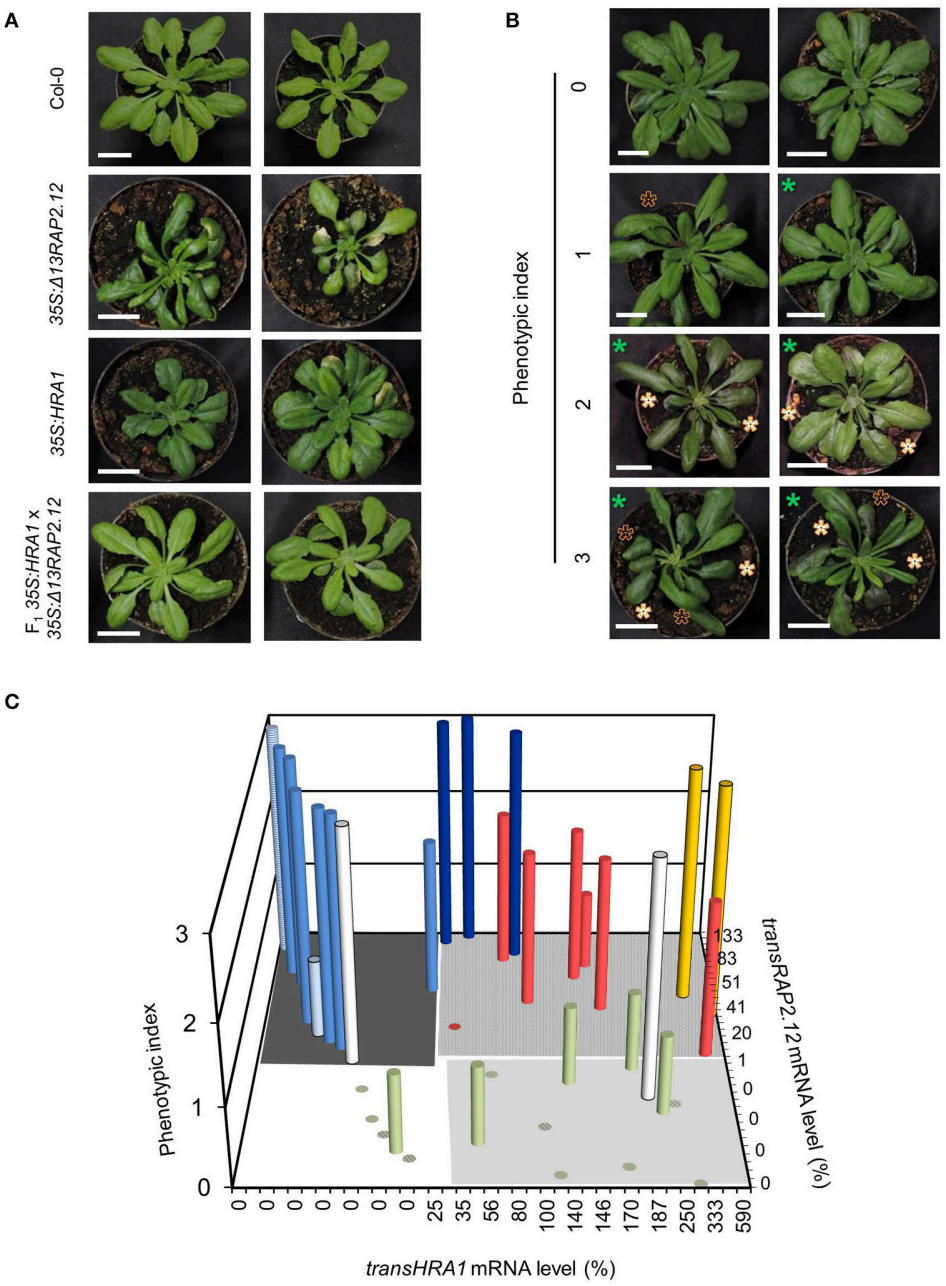
**Modulation of the Δ13RAP2.12 phenotype by high levels of *HRA1* expression. (A)** Representative morphology of parental *35S:*Δ*13RAP2.12* and *35S:HRA1* plants, first generation hybrids and wild type Col-0 plants at the adult stage of rosette development. Scale bar = 2 cm. **(B)** Sample output of the visual ranking procedure applied for the phenotypization of the F_2_ population. Appearance of the glossy leaves feature is marked with white asterisks, curved leaves are indicated with black ones, while smaller plant diameter can be inferred from the scale bar (2 cm) and is marked by green asterisks. **(C)** Bar plot of phenotypic index as a function of *transRAP2.12* mRNA abundance and presence of transgenic *HRA1*. Each column represents an F_2_ individual, or a plant of a reference genotype (hatched columns). *transRAP2.12* and *transHRA1* were expressed as percent relatively to one *35S:*Δ*13RAP2.12* or *35S:HRA1* parental plant, respectively. Support data for the diagram can be found in Table 2. The grouping of the bars in different colors is discussed in the main text.

We chose to investigate the effects of HRA1 on the transcriptional activity of RAP2.12 by crossing homozygous *35S:*Δ*13RAP2.12* and *35S:HRA1* parental plants. The former parental genotype expressed an N-terminally mutated form of RAP2.12 that, by escaping the oxygen-dependent degradation, allowed us to study a constitutive hypoxic response in plants kept in aerobic conditions (Licausi et al., 2011b). Constitutive expression of an oxygen-insensitive RAP2.12 form leads to widespread morphological changes in arabidopsis (Weits et al., 2014). *35S:*Δ*13RAP2.12* plants developed abnormal lateral organs in the vegetative rosette, where leaves often displayed irregular margins, bent and twisted petioles associated with downwards curling leaves, and enhanced wax deposition that results in higher stiffness and glossy appearance. Bleaching and necrosis of leaves was also commonly observed in this parental line (Figure 1A).

In this genetic background, we assessed whether and to which extent the over-expression of HRA1 modulated the activity of RAP2.12. We analyzed the F_2_ progeny of the cross, which, as a segregating population, enabled us to observe the combinatorial effect of the two loci in a uniform genetic background. Among 32 F_2_ plants, the expression of the transgenes ranged from 0 to 133% for Δ*13RAP2.12* (indicated as *transRAP2.12*) and from 0 to 590% for *HRA1* (*transHRA1*), as compared to a reference parental plant whose expression was set at 100% (Table 2). Variable transgene expression levels could be explained by the segregation of the two T-DNAs, as well as by an intrinsic degree of individual variation derived from the parental lines.

**Table 2:** **Expression values of the two transgenes and phenotypic index value of the plants used in the analysis**.

**Plant name**	**transHRA1 (%)**	**transRAP2.12 (%)**	**Phenotypic index**	**Assigned genotype**
wild type#1	0	0	0	Wild type
F_2_#1	0	0	1	
F_2_#2	0	0	0	
F_2_#3	0	0	0	
wild type#2	0	0	0	
F_2_#4	0	1	3	*35S:Δ13RAP2.12*
F_2_#5	0	9	3	
F_2_#6	0	19	3	
F_2_#7	0	20	1	
F_2_#8	0	34	3	
F_2_#9	1	48	2	
F_2_#10	0	51	3	
F_2_#11	0	65	2	
35S:Δ13RAP2.12	0	100	3	
F_2_#12	35	0	1	*35:HRA1*
F_2_#13	56	0	0	
35:HRA1#1	100	0	0	
F_2_#14	122	0	0	
F_2_#15	140	0	1	
F_2_#16	170	0	0	
F_2_#17	182	0	1	
F_2_#18	187	0	3	
F_2_#19	196	0	1	
35:HRA1#2	250	0	0	
F_2_#20	275	0	0	
F_2_#21	25	109	3	*35S:Δ13RAP2.12, 35:HRA1*
F_2_#22	26	25	0	
F_2_#23	35	133	3	
F_2_#24	72	83	2	
F_2_#25	80	90	3	
F_2_#26	82	43	2	
F_2_#27	120	58	2	
F_2_#28	146	77	1	
F_2_#29	161	41	2	
F_2_#30	333	44	3	
F_2_#31	362	6	2	
F_2_#32	590	38	3	

As a first remark, the strong phenotype displayed by the *35S:*Δ*13RAP2.12* parental was attenuated in the F_2_ population, which showed a variable extent of reversion to the wild type phenotype. This observation prompted us to look for a correlation between abundance of the two transgene products and phenotypic aspect of the plants. We ranked the phenotypes displayed by the hybrid progeny by means of three main qualitative hallmarks of the Δ13RAP2.12-related morphology: smaller rosette (parameter P1), petiole bending (P2), and increased waxiness of leaf adaxial surfaces (P3). The evaluation of each qualitative parameter describing the Δ13RAP2.12-related phenotype was carefully made. For P1, smaller rosette size had to be coupled with normal petiole and leaf blade length, to avoid confusion with the *35S:HRA1* phenotype (compact rosette with contracted petioles and rounder leaf shape (Figure 1A and Giuntoli et al., 2014). For P2, bending was scored when it coincided with altered leaf margin shape and curling of the leaf blade. In parameter P3, finally, the presence of leaf gloss and enhanced thickness were both required. The presence or absence of each parameter was scored upon visual inspection and expressed as a binary value (0 or 1), the three scores were summed and each plant's phenotype was expressed by a lumped index ranging from 0 (near-wild type morphology) to 3 (near-Δ13RAP2.12 morphology) (Figure 1B).

The first hybrid generation presented a uniform morphology with intermediate Δ13RAP2.12 traits, namely bent and curled leaves with rounder blades (Figure 1A). Such outcome might be due either to an incomplete dominance of the *transRAP2.12* allele in the heterozygous configuration, or to a functional balancing between *transRAP2.12* and *transHRA1* alleles. In the subsequent F_2_ generation, the observed phenotypes segregated and their distribution was plotted against the expression level of the two transgenes (Figure 1C and Table 2).

With two exceptions (yellow columns), top phenotype index scores were assigned to plants that expressed *transRAP2.12* alone (light blue columns, *transRAP2.12* = 9–100%) or to such an extent that *transHRA1* expression could be overcome by RAP2.12 (dark blue columns, *transRAP2.12* = 90–133%). The absence of transgenic Δ*13RAP2.12* expression, on the other hand, translated into a low phenotype index (olive green columns). Only one plant showed low phenotype in spite of detectable *transRAP2.12* expression and absence of the *HRA1* transgene (pale light blue column, *transRAP2.12* = 25%), indeed all RAP2.12 targets analyzed in the subsequent gene expression analysis proved to be lowly expressed for this individual, hinting at a reduced activity of the stabilized transcription factor as the cause of the phenotype in this plant. On the opposite, two plants (white bars) presented a strong phenotype, in front of very low *transRAP2.12*) or undetectable *transRAP2.12* expression; even assuming *transRAP2.12* to be already active in the first case, we could not explain the observed phenotype in the second. In all the remaining plants (8/32, red columns), concurrent *HRA1* over-expression was able to restrict the impact of Δ*13RAP2.12* expression (phenotype index = 0–2, *transRAP2.12* = 6–83%), defining the borders of a RAP2.12-HRA1 balancing zone in our diagram. An average phenotypic index, obtained as the sum of the index of all plants in a set divided by their number, passed from 2.6 in plants only expressing *transRAP2.12* (dark gray-shaded quarter in the diagram in Figure 1C, *transHRA1* = 0–1%) to 2.2 in the set of plants also expressing *transHRA1* (light gray-shaded quarter, *transHRA1* >25%), while it reached 0.2 in wild type plants (white quarter) and 0.6 when only *transHRA1* was expressed (pale gray-shaded quarter). Overall, we consider this assessment in favor of the hypothesis that abundant HRA1 protein could contrast the activity of the oxygen-insensitive version of RAP2.12, assumed as correlated to the phenotype index in *35S:*Δ*13RAP2.12* plants.

### Activation of RAP2.12 target genes is affected by HRA1 *in planta*

After assessing the impact of HRA1 on the Δ13RAP2.12 phenotype, we moved forward and analyzed the impact in terms of molecular markers. RAP2.12 stabilization and over-expression is known to cause constitutive expression of core hypoxia-responsive genes in arabidopsis (Licausi et al., 2011b). Therefore, we considered appropriate to evaluate the correlation between *RAP2.12* over-expression and expression of hypoxic targets in our F_2_ population, and verify to which extent it might be affected by *HRA1* over-expression. The set of marker genes included in the analysis encompassed the transcription factor *LBD41* (*At5g02550*), the acyl-CoA desaturase *HUP7* (*At1g43800*), the cysteine oxidase *PCO1* (*At5g15120*), the two fermentative genes *PDC1* (*At4g33070*) and *ADH* (*At1g77120*), the non-symbiontic hemoglobin *HB1* (*At2g16060*), and *HRA1* (*At3g10040*) itself. The anoxia-responsive heat shock factor *HSP18.2* (*At5g59720*) was, moreover, selected as a negative control gene, since a survey of public trascriptomic data suggested it not to be activated in the N-end rule mutant backgrounds *ate1/2* and *prt6* (Pucciariello et al., 2012).

**Figure 2:**
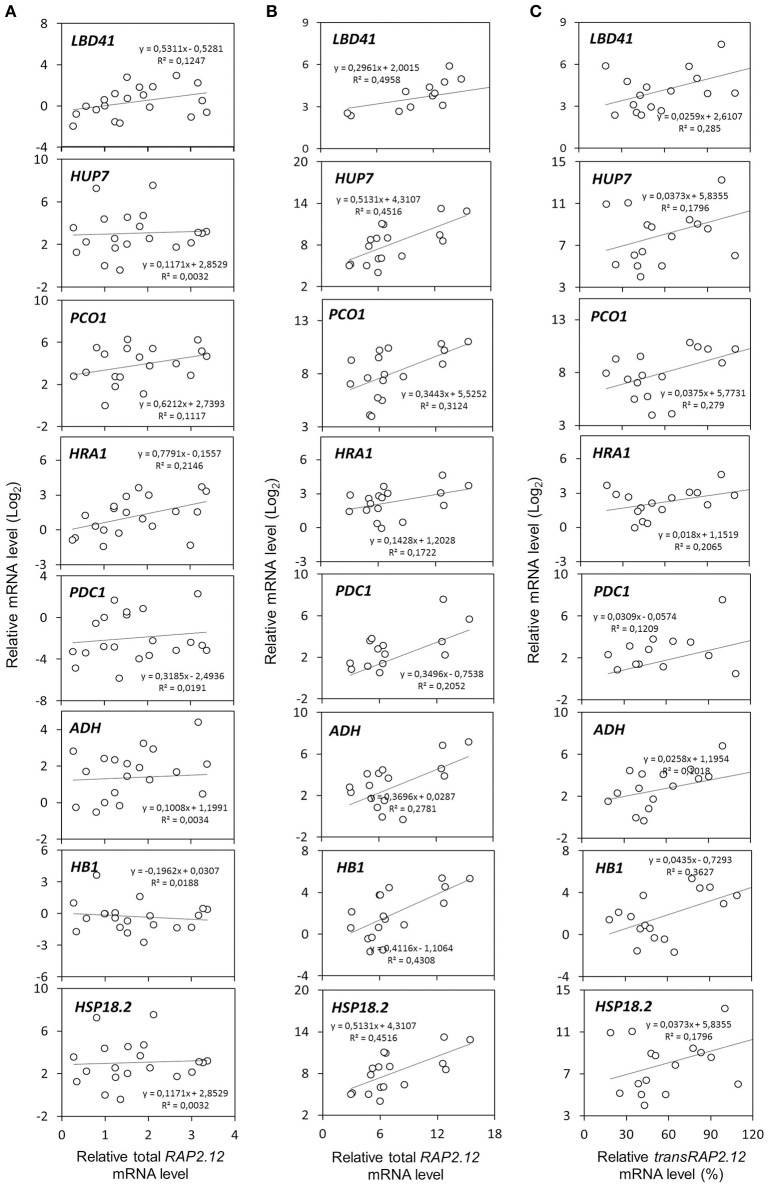
**Correlation between *RAP2.12* and target gene expression**. Linear regressions of anaerobic mRNA levels in F_2_ (*n* = 32), parental (*n* = 3), and Col-0 plants (*n* = 2). The full set of plants was split into two subsets, based on **(A)** the absence (*n* = 16) or **(B,C)** presence of *transRAP2.12* transgene expression (*n* = 21). For the latter subset of plants, expressing the Δ13RAP2.12 T-DNA, target gene expression was correlated either with total *RAP2.12* levels **(B)** or *transRAP2.12* transgene levels **(C)**. For the former, total *RAP2.12* expression corresponded to the endogenous *RAP2.12* transcript **(A)**. Every dot represents an individual plant.

Two populations of transcripts corresponding to *RAP2*.12 were quantified and correlated to target gene expression. The mRNA encoded by the Δ*13RAP2.12* transgene was measured with *transRAP2.12* specific primers, while total *RAP2.12* expression represented the cumulative amount of the transcript encoded by the endogenous genomic locus and by the transgene, when present. Predictably, total *RAP2.12* expression displayed poor correlation with the aerobic levels of the targets in plants lacking Δ*13RAP2.12* transgene expression (Figure 2A), where the endogenous *RAP2.12* mRNA was translated in the oxygen-labile, inactive form of the transcription factor. Instead, a closer relationship was found in Δ*13RAP2.12* over-expressors, when target transcript levels were plotted either against total *RAP2.12* (Figure 2B) or *transRAP2.12* expression values (Figure 2C). Total *RAP2.12* was, therefore, assumed as a suitable predictor of target expression and kept for the subsequent analysis. Incidentally, *HSP18.2* proved to be activated by Δ13RAP2.12 in a similar fashion to the other well-known core hypoxia-responsive genes and was, therefore, assimilated to the other target genes.

**Figure 3:**
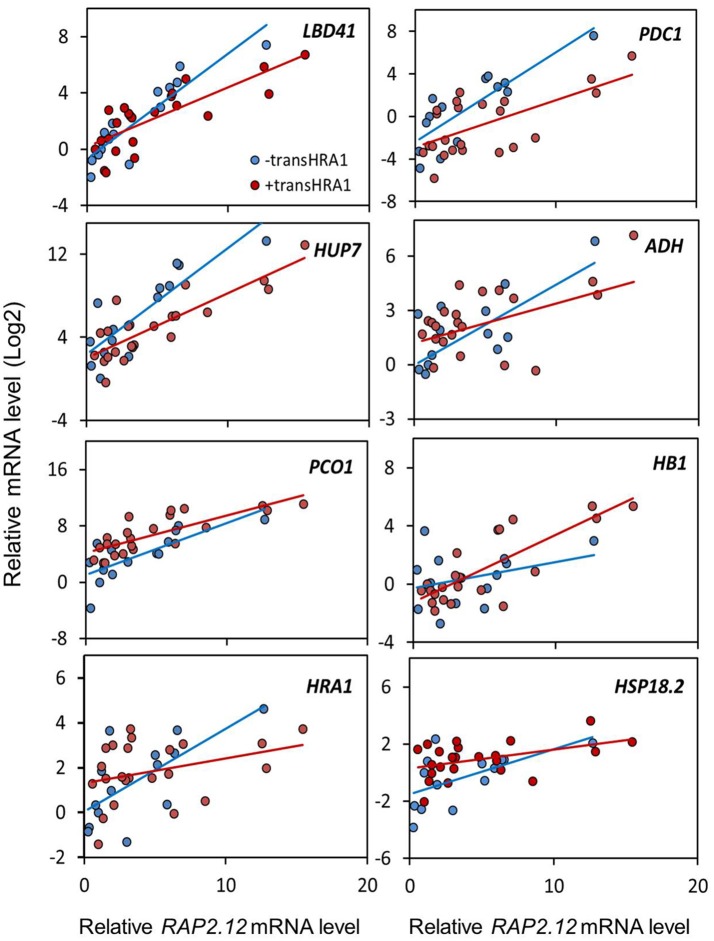
**Effect of *HRA1* over-expression on RAP2.12 targets**. F_2_ (*n* = 32), parental (*n* = 3) and Col-0 plants (*n* = 2) were clustered into two groups, distinguishing *HRA1* over-expressors (“+transHRA1,” *transHRA1* >25%, *n* = 23) from plants with wild type *HRA1* levels (“-transHRA1,” *transHRA* = 0–1%, *n* = 14), and the expression of the targets was plotted against total *RAP2.12* expression.

To assess the effect of HRA1 on the RAP2.12-mediated target activation (Figure 2) the F_2_ population was split into plants over-expressing *HRA1* (“+transHRA1” plants) and those not (“-transHRA1”). With an ANCOVA, the tendency of HRA1 to limit RAP2.12 activation power on the anaerobic targets was measured. A linear model was fit to the scatterplot expression of every RAP2.12 target (Figure 3), using total *RAP2.12* expression values and *transHRA1* presence as predictors (Table 3). The analysis confirmed the existence of a significant effect exerted by total *RAP2.12* expression over the steady state mRNA levels of all selected markers and furthermore highlighted a contribution by *HRA1* transgene expression (Table 4). HRA1 importance was especially apparent from the significant interaction terms between the two predictor variables in the case of *LBD41, HUP7*, and marginally for *HB1, HRA1*, and *PDC1*. However, no HRA-RAP2.12 interaction occurred for *ADH, HSP18.2*, and *PCO1* activation. In four of the five cases where the interaction took place, the expression of *transHRA1* had an antagonistic effect in respect to *RAP2.12*, as indicated by the negative coefficients for the “RAP2.12 x transHRA1” term in the linear model (Table 3).

**Table 3:** **Summary of linear model parameters**.

**Gene**	**Intercept**	**Coefficient (RAP2.12)**	**Coefficient (transHRA1)**	**Coefficient (RAP2.12 x transHRA1)**
*LBD41*	−0.48 ± 0.37	0.52 ± 0.07	0.59 ± 0.47	−0.23 ± 0.09
*HUP7*	1.62 ± 0.53	0.71 ± 0.1	−0.28 ± 0.67	−0.28 ± 0.13
*PCO1*	0.74 ± 0.54	0.51 ± 0.11	2.15 ± 0.69	−0.14 ± 0.13
*HRA1*	0.03 ± 0.37	0.26 ± 0.07	0.88 ± 0.48	−0.18 ± 0.09
*ADH*	0 ± 0.78	0.44 ± 0.16	1.22 ± 1.00	−0.22 ± 0.19
*PDC1*	−1.67 ± 0.60	0.59 ± 0.11	−0.33 ± 0.77	−0.28 ± 0.14
*HB1*	−0.28 ± 0.43	0.13 ± 0.09	−0.65 ± 0.56	0.19 ± 0.10
*HSP18.2*	−1.05 ± 0.36	0.22 ± 0.07	1.25 ± 0.47	−0.13 ± 0.09

**Table 4:** ***P*****-values from the ANCOVA of RAP2.12 target gene expression**.

**Gene**	**p (RAP2.12)**	**p (transHRA1)**	**p (RAP2.12 x transHRA1)**
*LBD41*	***		*
*HUP7*	***	**	*
*PCO1*	***	**	
*HRA1*	**		°
*ADH*	**		
*PDC1*	***	**	°
*HB1*	***		°
*HSP18.2*	***	*	

ANOVA summary tables were calculated for each RAP2.12 target upon generation of separate linear models. The predictors used were total RAP2.12 expression (continuous variable) and HRA1 over-expression (categorical variable). Symbols indicate statistical significance in the intervals specified by the following legend: 0 “***” 0.001 “**” 0.01 “*” 0.05 “°” 0.1.

We conclude that the assessment of RAP2.12 transcriptional activity, estimated from the expression of established marker genes, was in substantial agreement with the prior evaluation of its ability to affect plant morphology, when made stable in air and over-expressed. Broadly speaking, both pieces of evidence we collected, indeed, pointed at the ability of HRA1 to restrict RAP2.12 functionality, although the extent of HRA1 impact seems confined to a precise range of RAP2.12 protein abundance.

## Discussion

Tight regulation of gene expression to withstand fluctuations in the intracellular oxygenation status is likely to be vital for organisms, like the terrestrial plants, that have not evolved specific systems for capillary oxygen delivery. In *A. thaliana*, transcription in response to low oxygen signals is redundantly triggered by the homologous ERF-VII transcription factors RAP2.2 and RAP2.12 (Hinz et al., 2010; Gibbs et al., 2011; Licausi et al., 2011c; Bui et al., 2015; Gasch et al., 2016). In our previous work, we have found evidence that the HRA1 transcription factor, whose constitutive expression in arabidopsis leads to marked reduction of hypoxic responses in oxygen-deprived plants, interacts with RAP2.12 and, in doing so, carries out a counterbalancing effect on the activation of RAP2.12 hypoxic target genes (Giuntoli et al., 2014). The presented research aimed at showing to which extent the hypoxic response attenuator HRA1 is effective *in planta* in modulating the transcription of RAP2.12 target genes and the production of phenotypes associated with RAP2.12 over-expression in arabidopsis. Previous demonstrations of the impact of this TF interaction are related to the response of isolated arabidopsis protoplast cells. Here, we combined the ectopic expression of HRA1 with that of an oxygen-insensitive form of RAP2.12, which enabled us to investigate the regulation of the anaerobic response without imposing external stress conditions on plants. Deployment of a segregating population made the correlation possible, in each individual plant, between the actual expression level of both transgenes, which spanned a range of combinations, and two marker traits describing RAP2.12 activity.

With this approach, we were able to spot the balancing action exerted by HRA1 on RAP2.12 by examining the phenotype of plants growing in normal conditions. Initial clues of the efficacy of such a mechanism in fully developed, unstressed plants had appeared previously, with the observation that stable transformation of *35S:HRA1* plants with a *35S:*Δ*13RAP2.12* T-DNA generated a progeny in which the phenotypic traits associated to *HRA1* over-expression reverted to the wild type (Giuntoli et al., 2014). The achievement of comparable outcomes following two independent events of T-DNA insertion in the genome supports the conclusion that a causal link subsists between concurrent over-expression of *HRA1* and reversion of the molecular and phenotypic effects of Δ13RAP2.12.

Furthermore, the present study provides the first quantitative description of RAP2.12-HRA1 balancing in whole developed plants, evaluated by means of anaerobic molecular markers. As highlighted before, this result was achieved by over-expression of the two transcription factors under control of the constitutive CaMV 35S promoter. Despite this simple strategy, whereby massive accumulation of either protein is allowed unrestrictedly during the entire plant lifespan, functional balancing proved to be still in place and amenable to quantitative modeling.

While analyzing plant responses, we decided to reconstruct the behavior of HRA1 as a function of the presence or absence of its expressed transgene (Figure 3), as quantified through specific qPCR amplification. This is because, in first place, total *HRA1* transcript levels were so superior in the over-expressing plants (Log_2_ *HRA1* = 10.2–13.8; values refer to the expression measured in one of the wild type reference plants, taken as reference and set to Log_2_ *HRA1* = 0), as compared to those detected in plants with wild type *HRA1* and *RAP2*.12 configuration (Log_2_ *HRA1* = −1.2–2.6), or in Δ*13RAP2.12* over-expressing plants (Log_2_ *HRA1* = 3–5.8), that approximation to a categorical condition was allowed. In second place, we assumed that *HRA1* mRNA steady state level could be *bona fide* considered as proportional to protein abundance, in the absence of any known mechanism of targeted post-transcriptional regulation specific for this gene. Therefore, a model where the HRA1 transcription factor was approximated as highly abundant, as in *35S:HRA1* individuals, or lowly abundant, as in all other genotypes, was considered acceptable to account for the balancing effect. The same did not apply to *RAP2.12*, which required treatment as a continuous quantity. A linear increase of marker gene expression was recorded with increasing total *RAP2.12* (or *transRAP2.12*) abundance along the range of expression available in our measurements for this predictor (Figures 2B,C), suggesting that the over-expressed Δ13RAP2.12 protein was not abundant enough to saturate the target promoters.

Our investigation took advantage of striking phenotypic features that associate to the ectopic expression of an oxygen-insensitive variant of RAP2.12. Unraveling the downstream events that realize this specific ontogenetic program was beyond the aim of this work and might be worth focused investigation. Nonetheless, we might conclude that the phenotype originates from the accumulation of Δ13RAP2.12, rather than from spurious phenomena due to the untargeted process of T-DNA integration, because it can be at least partially rescued by the expression of a RAP2.12-specific repressor, HRA1. In the same way, the phenotypic consequences of *35S:HRA1* expression could be considerably reverted by enhancing RAP2.12 activity (this study and Giuntoli et al., 2014). Therefore, it is reasonable to think that the alterations visible in the Δ13RAP2.12 phenotype are caused by genes differentially regulated by *35S:*Δ*13RAP2.12* and subjected to HRA1-dependent negative regulation.

The interplay between HRA1 and RAP2.12 was revealed by the expression of transcriptional markers, namely known plant hypoxic targets identified from the broad specialized literature. Marginally, it could be noticed that our analysis provided further confirmation to the fact that aerobic transcription of *RAP2.12* results in inactive protein accumulation. It has been noticed before that constitutive expression of the full version of RAP2.12 leads to minimal up-regulation of anaerobic gene expression in air and does not cause any detectable plant phenotype (Licausi et al., 2011b). Coherently, we found limited correlation between full-length *RAP2.12* mRNA levels and RAP2.12 transcriptional targets (Figure 2A). Beside this, the ANCOVA highlighted a different degree of specificity in the targets we considered. More specifically, while some anaerobic genes are exclusively regulated by RAP2.12, cross-talk from different cell pathways is known to converge on other core hypoxia-responsive genes. Indeed, RAP2.12 function is known to be superimposed on unrelated regulatory pathways, such as the one brought about by heat shock factors on *HSP18.2* (Nishizawa et al., 2006; Guo et al., 2008) and multiple ABA-mediated signaling events influencing *ADH* expression (de Bruxelles et al., 1996; Xiong et al., 2001; Papdi et al., 2015). We speculate that this might explain why the HRA1-mediated repression of RAP2.12 was not detectable on *ADH* and *HSP18.2* (Table 4), being any additional regulation beyond the predictive power of our bifactorial model. Detailed promoter survey of representative genes for the two regulatory classes, aided by the recent identification of the *cis*-element recognized by RAP2.12 in its target promoters (Gasch et al., 2016), might unveil the near-exclusive presence of the RAP2.12-specific binding site in the first class of items and support our hypothesis.

In this first report of the effective balancing between HRA1 and RAP2.12 in the aerial tissues of arabidopsis, the equilibrium of the two transcription factors was moved to a non-physiological range, by deployment of over-expression constructs. Future steps of this research might take advantage of native gene promoters to understand whether, under physiological expression conditions, the transcriptional complex is actually able to modulate target gene expression by originating transient transcriptional responses.

## Author contributions

BG, FL, and PP designed the experiments that were carried out by BG and FL. HvV, and BG performed the statistical analysis. BG, wrote the manuscript. FL, HvV, and PP critically revised it.

## Funding

Results have been achieved within the framework of the 1st call ERA-NET for Coordinating Plant Sciences, with funding from Scuola Superiore Sant'Anna.

### Conflict of interest statement

The authors declare that the research was conducted in the absence of any commercial or financial relationships that could be construed as a potential conflict of interest.
